# Cerebral Organoids—Challenges to Establish a Brain Prototype

**DOI:** 10.3390/cells10071790

**Published:** 2021-07-15

**Authors:** Artem V. Eremeev, Olga S. Lebedeva, Margarita E. Bogomiakova, Maria A. Lagarkova, Alexandra N. Bogomazova

**Affiliations:** 1 Federal Research and Clinical Center of Physical-Chemical Medicine of Federal Medical Biological Agency, Malaya Pirogovskaya, 1a, 119435 Moscow, Russia; oslebedeva@rcpcm.org (O.S.L.); margbog_5@mail.ru (M.E.B.); lagar@rcpcm.org (M.A.L.); abogomazova@rcpcm.org (A.N.B.); 2 Center for Precision Genome Editing and Genetic Technologies for Biomedicine, Federal Research and Clinical Center of Physical-Chemical Medicine of Federal Medical Biological Agency, Malaya Pirogovskaya, 1a, 119435 Moscow, Russia

**Keywords:** iPSCs, brain organoids, differentiation, electrophysiology, neurotrophic factors

## Abstract

The new cellular models based on neural cells differentiated from induced pluripotent stem cells have greatly enhanced our understanding of human nervous system development. Highly efficient protocols for the differentiation of iPSCs into different types of neural cells have allowed the creation of 2D models of many neurodegenerative diseases and nervous system development. However, the 2D culture of neurons is an imperfect model of the 3D brain tissue architecture represented by many functionally active cell types. The development of protocols for the differentiation of iPSCs into 3D cerebral organoids made it possible to establish a cellular model closest to native human brain tissue. Cerebral organoids are equally suitable for modeling various CNS pathologies, testing pharmacologically active substances, and utilization in regenerative medicine. Meanwhile, this technology is still at the initial stage of development.

## 1. Introduction

In previous studies of the nervous system development and function in health and human pathologies, the following models have been used: animal models in vivo [1,2,3,4,5]; intravital sections of the brain ex vivo [6,7,8]; primary neuronal culture of cells or brain tissue in vitro [9,10,11,12,13], and, to a lesser extent, post-mortem material [14,15,16,17,18]. All these experimental methodological approaches have their limitations. For example, most cognitive ability studies cannot be carried out on animal models [19]. Additionally, animal models have limitations in testing pharmacological drugs that affect behavior and learning [20]. Animal models are not always suitable for electrophysiological mapping of neurons, modeling neural networks, and developing neuron interaction algorithms with various electronic devices for medicine [21]. Intravital brain slices are sensitive to axotomy [22]. Consequently, despite the preservation of native connections between cells, neuronal network activity can be distorted by the death of neurons [23]. The use of 2D neuronal tissue cultures allows studying the fundamental cellular, metabolic, and electrophysiological mechanisms of neural networks. However, the structure of 2D neuronal tissue culture differs from nervous tissue in vivo. It does not have the proper microenvironment and architecture of the nervous system [24]. The rapid loss of cell viability limits post-mortem slice material use. Great hopes for overcoming the above models’ imperfections are associated with developing a technology for obtaining 3D structures of nervous tissue using differentiated derivatives of iPSCs, the so-called cerebral organoids.

### 1.1. A Brief History of Cerebral Organoid Technology

#### 1.1.1. Early Observations of Cell Self-Organization

The history of organoid technology began more than a century ago. A pioneering study in this area is Henry Wilson’s work, which demonstrated that sea sponges, dissociated into individual cells, can re-unite into fully functional organisms [25]. In the 1950s and 1960s, many works showed that cells dissociated from complex organisms could re-aggregate into organized structures. In 1955, Steinberg and Gilbert [26] reported that early amphibian embryos dissociated to single cells could reassemble into spatially oriented segments of germ layers. In 1970, DeLong et al. discovered spontaneous aggregation of single-cell suspension of fetal mouse hippocampal neurons into aggregates with a histological pattern resembling normal hippocampal architecture [27]. Reinwald and Green successfully created 3D aggregates from human epidermal keratinocytes and irradiated mouse 3T3 fibroblasts [28]. The resulting aggregates consisted of a layer of proliferating cells and a layer of constantly differentiating keratinocytes, giving rise to the stratum corneum.

#### 1.1.2. The Emergence of iPSC-Based Protocols for Organoids

This field has progressed rapidly with emerging technology for embryonic stem cells (ESCs) and iPSCs [29,30,31,32], so-called pluripotent stem cells (PSCs). These cells have been shown to differentiate and form ordered structures depending on various growth factors and cellular microenvironment [33,34]. Over the past decade, many protocols have been published on creating brain organoids based on PSCs’ ability to self-organize and differentiate [35,36,37,38]. As organoids grow and mature, they can imitate human brain tissue 3D structure [39,40,41,42,43]. Various methodological approaches traditionally used to characterize intravital brain slices and 3D tissue structures in vitro have been adapted, upgraded, and applied to study brain organoids. For example, visualization methods have been improved [38,44], and new image processing algorithms have been created [45]. The genetic modifications of organoid cells using viral systems and synthetic oligonucleotides have been applied to study pathologies of brain development [46].

The existing modulation methods of the current/voltage strength were adapted to study electrophysiology [47]. Some studies used multielectrode arrays (MEA) for monitoring the electrical activity in organoids [40,48]. Storm et al. have optimized methods for visualization of calcium [49] in organoids, enabling the following of the intercellular and intracellular processes underlying neuron electrophysiology and showing synaptic connection presence. The authors predict the oxygen gradients in brain organoids using computer simulations [50].

#### 1.1.3. The iPSC-Based Organoids for Disease Modeling

A rapidly growing number of studies model various brain pathologies and test potentially promising therapeutic agents in organoids. For example, the pioneering work on cerebral organoids conducted using iPSCs of a patient with microcephaly caused by a mutation in the CDK5RAP2 gene [38]. The neuronal progenitor cells of the “diseased” organoid had low proliferative activity and differentiated faster than in the control organoid. Knockout of the CDK5RAP2 gene in healthy iPSCs resulted in a similar phenotype in organoids. In another study, brain organoids were established from iPSCs of patients with genetically determined epilepsy [51], making it possible to study the electrophysiological activity of “diseased” neurons. Birey et al. (2017) created organoids from iPSCs with point mutations in the CACNA1C gene, causing Timothy syndrome and associated with a nervous system disorder [52]. The atypical behavior of cells was demonstrated in “mutant” organoids. They also showed the possibility of correcting the mutation using therapeutic agents, an *L*-type calcium channel inhibitor (nimodipine). Conforti et al. used the same approach for the study of Huntington’s disease [53]. The work showed changes in proliferation and differentiation of neurons, which would be impossible to investigate in patients with such pathology. In studies of the hereditary form of Parkinson’s disease caused by a mutation in the LRRK2 gene, the resulting organoids contained a reduced number of dopaminergic neurons. Additionally, they had a disease-relevant phenotype [54,55]. LRRK2 kinase inhibitors were tested as potential agents for the therapy of Parkinson’s disease in such organoids [56]. Recent studies in brain organoids with the phenotype of Alzheimer’s disease showed accumulation of Aβ and tau peptides [57]. Brain organoids have also been successfully used to study several infectious diseases causing damages to the nervous system. For instance, transcriptome analysis of brain organoids infected with the Zika virus allowed identifying cellular signal transmission pathways during viral infection [58]. Moreover, brain organoids can be used for screening drugs intended for the treatment of viral infections in humans [59]. In particular, Song et al. (2020) showed that the SARS-CoV-2 virus causes damage to the central nervous system depending on the presence of ACE2 receptors [60]. Cerebral organoids may also help in cancer research because they can better imitate the tumor structure and its microenvironment than a conventional 2D culture. For instance, in the organoid model of glioblastoma [61], cancer stem cells were located at the organoid periphery, exhibiting a high renewal rate. In contrast, those located in the central part of the organoid were dormant and senescent [62]. Glioblastoma cells in organoids showed higher resistance to chemotherapy and the effects of ionizing radiation than in 2D culture [63].

#### 1.1.4. The Therapeutic Potential of Brain Organoids

Some works showed that cerebral organoids have the potential for cell therapy of central nervous system diseases. Mansour et al. (2018) [46] labeled 40–50-day old cerebral organoids with GFP by viral transduction and implanted them into the brains of 5–6-week-old NOD/SCID mice. Using immunohistochemical methods, the authors detected the grafted organoids in the mouse brain by the presence of cells positively stained for SOX2, PAX6, and GFAP, the “human” axons in other areas of the brain, as well as the presence of CD31 positive mouse cells in blood vessels that have grown into an organoid. Optogenetic methods have shown a functional synaptic connection between transplanted organoids from human cells and mice brains. Daviaud et al. obtained similar results in comparative experiments on transplantation of neuronal progenitor cells and brain organoids made from human iPSCs expressing GFP into mice brains. The authors demonstrated the vascularization of organoids and their further maturation [64]. Additionally, several laboratories reported the first experiments to develop therapeutic organoids in animal models [65,66,67].

#### 1.1.5. Current Challenges for Brain Organoid Technology

Meanwhile, despite the growing use of organoid technology in recent years, many problems still must be addressed. It is not currently possible to create standardized organoids with a specific and reproducible cellular composition, which would have the characteristics of mature native nervous tissue (higher-order functions: electrophysiological activity, the ability to form neural networks with electrical activity) [41]. Another equally important problem is developing an enviable central part in organoids in the process of long-term cultivation, in which cells die due to the hindered diffusion of oxygen and nutrients [50]. Comparative studies of transcriptome profiles of human brain tissues and organoids have revealed both many similarities and significant differences [68,69,70,71]. In particular, the most significant similarity is between “immature” organoids and early embryonic brain tissues. Both structures use the same genes, which control the proliferation and differentiation of progenitor cells. However, there is an apparent difference between organoids and fetal tissues of late development stages. We hypothesize that more prolonged organoid cultivation may improve maturation, bringing them closer to the fetal brain tissues.

Therefore, today, there is a need for inexpensive protocols for obtaining highly viable brain organoids standardized in terms of culture time, structure, and functional activity. Below we consider the main features of the protocols for obtaining brain organoids, the main problems of this technology, and the search for problem solutions.

## 2. The First Protocols for Creating Brain Organoids from PCSs

The production of organoids from PSCs is the most exciting and promising technology for developing physiologically relevant in vitro models of the human brain. The impetus for developing the technology was the publication of the microcephaly study by Lancaster et al. [38] and Kadoshima et al. [37]. Even though these protocols are rather laborious and do not allow scaling, they became basic protocols for subsequent works.

### 2.1. Lancaster Protocol

The first stage of the Lancaster et al. protocol is the initiation of nonspecific pluripotent cells differentiation into embryoid bodies containing the derivatives of three germ layers: endoderm, mesoderm, and ectoderm. For this, the single-cell suspension of PSCs is placed into round-bottom wells of a 96-well plate, promoting 3D cell aggregates formation. Reducing the concentration of bFGF in the PSC medium triggers cell differentiation. The resulting embryoid bodies are transferred into a medium with components promoting neuroectoderm formation. Subsequently, the organoids are cultured on paraffin films in drops of the neurotrophic medium mixed with Matrigel. Matrigel, which represents the extracellular matrix, creates a capsule around the future organoid, preventing morphogen diffusion, and makes gradients of their concentration inside the organoid. According to the authors, this stage contributes to forming a neuroepithelial layer with an internal cavity filled with fluid. At the end of the cultivation stage, 30–80% of organoids in drops have morphological features of neural epithelium. After that, the neurotrophic media is supplemented by retinoic acid serving as an additional agent for differentiation. The dynamic cultivation conditions are applied to improve the trophic of growing cerebral organoids. This protocol does not allow obtaining many organoids routinely, mainly due to the stage associated with incubation in micro drops on parafilm.

### 2.2. Kadoshima Protocol

The protocol, developed by Kadoshima et al., allows producing organoids of cortical neurons. As in the Lancaster protocol, for the transition to the 3D format, the authors use the sticking property of dissociated ESCs, which form spheroids during cultivation in low-adhesion 96-well plates [37]. In contrast to the Lancaster protocol, the authors combined switching to 3D format with triggering a specific differentiation of the PSC towards the terminal brain (telencephalon). They placed dissociated ESCs in a medium containing small molecules IWR-1-endo and SB-431542, inhibiting the WNT and TGFb signaling pathways, respectively. They removed inhibitors after 3 weeks of incubation. Then, the organoids were transferred to low-adhesion Petri dishes containing a neurotrophic medium and cultivated at an oxygen concentration of 40%.

### 2.3. Denham andand Dottori Protocol

Both PSCs and neuronal precursors can form 3D aggregates; therefore, in some protocols, the transition to the 3D format is carried out after the first stages of differentiation to neurons.

Denham and Dottori, in 2011, developed a method to produce neuronal progenitor cells through cultivation in neurospheres. The authors mechanically cut a colony of iPSCs into small pieces, which were previously induced to differentiate in the neuronal lineage. Then, they cultivated the pieces in the medium for pluripotent cells containing recombinant Noggin in a 96-well low-adhesion plate [72].

Nasr et al. (2018) modified the Denham and Dottori protocol to create cerebral organoids [73]. At first, the authors cut the ESC colonies to a size of 0.5 mm and then transferred them into a medium for neuronal differentiation and Petri dishes covered with laminin. For directed differentiation into dorsal forebrain neurons, the authors added small molecules SB431542 and LDN193189, inhibiting TGFb and BMP, respectively (double SMAD inhibition protocol). Two weeks later, to trigger the transition to the 3D format, the colonies were mechanically separated from the substrate and transferred to suspension culture. To stimulate the growth of organoids, the authors added growth factors bFGF and EGF to the medium. This method is challenging to standardize due to the stage of manual cutting of PSCs colonies.

**Figure 1:**
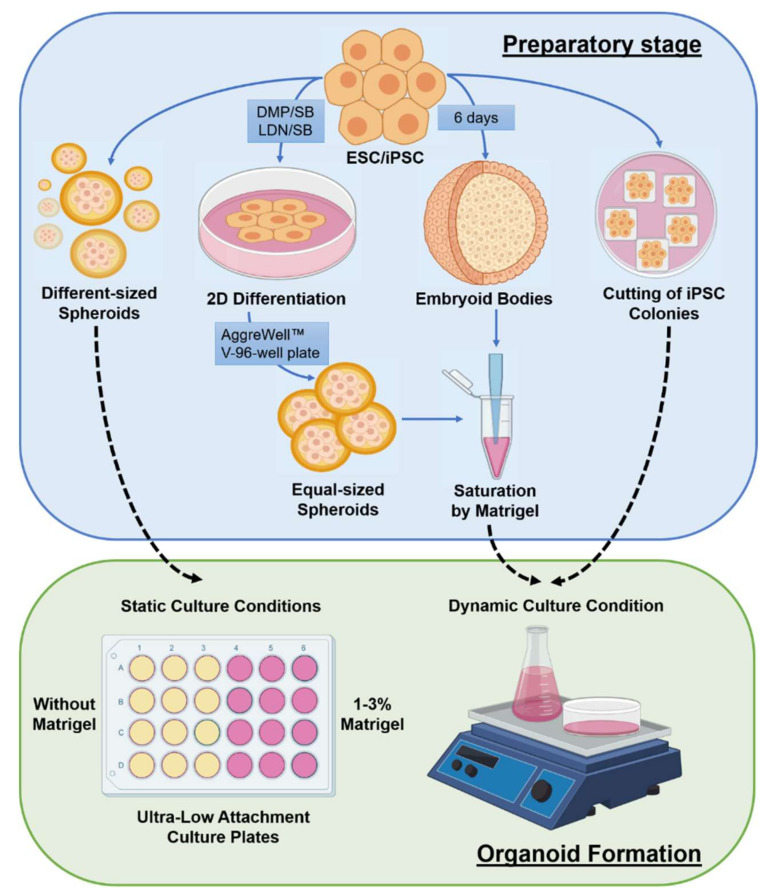
Basic strategies to produce cerebral organoids from PSCs. The preparatory stage includes various approaches for obtaining spheroids with or without preliminary differentiation into neuroepithelial progenitors. Then, the spheroids form cerebral organoids during long-term cultivation under stationary or dynamic culture conditions. DMP-dorsomorphin; SB- SB431542; LDN-LDN193189.

The protocols described above and their derivatives are primarily based on pluripotent or progenitor cell ability to self-organize and form a proper microenvironment. Usually, at the first steps, authors create spheroids, whose size is either not controlled or is determined by a fixed initial number of cells. Various neurotrophic factors, recombinant proteins and/or small molecules are also added to stimulate desirable and block unwanted developmental scenarios. For the growth and maturation of organoids, researchers cultivate them long-term, either in vessels with low adhesion or under dynamic conditions with continuous stirring (Figure 1).

## 3. The Specificities of Organoids Creation and Cultivation

### 3.1. Media and Components for Cultivation and Differentiation

**Figure 2:**
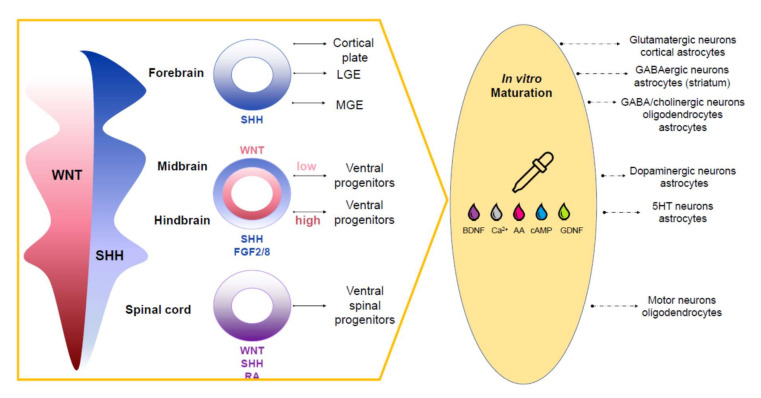
The gradient of morphogens, growth factors, and neurotrophic factors define the differentiation into a particular type of brain cells. Initially, the concentration of morphogens WNT and SHH is crucial. For example, the high concentration of SHH accompanied with a low level of WNT allows obtaining the forebrain structures. At the same time, the ventral distribution of the SHH concentration is also important—a high level allows the development of MGE zones; a low level allows for the cortical plate. High WNT at low SHH promotes the development of the spinal cord, and its dorsal gradient at low values, coupled with a high concentration of FGF2/FGF8, gives the development of ventral progenitors of the hindbrain, and in combination with an increase in the level of WNT, ventral progenitors of the midbrain develop. It is characteristic that for most types of neurons in all parts of the brain; the presence of neurotrophic factors, such as BDNF/GDNF, calcium ions, cAMP, and other factors, is necessary for maturation and functioning.

A culture medium and morphogenetic factors are crucial for forming and long-term cultivating cerebral organoids. A primary medium for the cultivation of neurons is DMEM/F12, which is rich in vitamins, amino acids, and other nutrients. There are also specialized neuronal culture media such as Neurobasal, Neurobasal-A (Thermofisher Scientific, Waltham, MA, USA), BrainPhys™ Neuronal Medium (StemCell Technologies, Vancouver, BC, Canada). Various substances for cultivating neurons in feeder-free and serum-free conditions are being used [74]: B27, a mixture of biotin, fat-soluble vitamins, albumin, hormones, enzymes involved in free radical metabolism [75], and/or a much poorer N2 supplement, providing survival and physiological activity of mature neurons [76]. Most studies use the B27 supplement to differentiate pluripotent cells toward the neuronal lineage combined with growth factors and morphogens: BDNF, TGFb, GDNF, FGF2/8, and retinoic acid [77,78]. To differentiate PSCs into neuronal precursors mimicking different parts of the brain, one needs to activate or suppress key signaling pathways under the control of morphogens SHH, WNTs, and growth factors BMP, FGFs, and SMAD. Notably, to obtain specific cells in organoids that mimic parts of the brain, the concentration gradient of SHH, WNTs, FGF2/8, and retinoic acid morphogens is crucial. Figure 2 demonstrates how varying concentrations of certain factors result in various brain structures.

For example, SHH at low concentrations leads to forming neural progenitors, which can then differentiate into the striatum dorsal GABAergic neurons [79]. In higher concentrations, SHH leads to the patterning of the ventral forebrain neuroepithelium and enables the formation of GABA interneurons and cholinergic neurons, precursors of the basal forebrain nuclei [80]. Small molecules, e.g., CHIR99021, activate the WNT pathway in a dose-dependent manner, forming neuroepithelium patterns in the forebrain, midbrain, and hindbrain [81]. The cost of small molecules has recently decreased, enabling the development of protocols for various brain cells. In addition to growth factors, low molecular weight substances can affect the maturation of neurons. For example, a gradual increase in the concentration of calcium and GABA ions promote the maturation of neurons which obtain pronounced synaptic activity through regulation of the CREB and cAMP pathways [82,83].

### 3.2. Necrotic Central Zones

**Figure 3:**
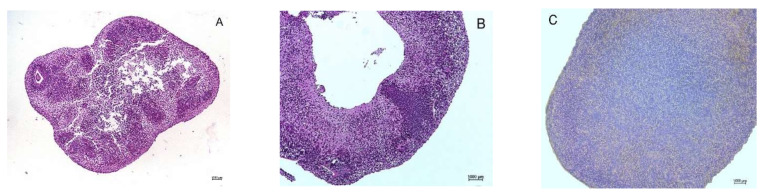
The central zone of cerebral organoids. (**A**) Organoid with a necrotic central zone. (**B**) The central zone degraded completely and disintegrated, forming a cavity. (**C**) Organoid with an intact central zone. Hematoxylin-eosin staining of cryosections fixed in 4% buffered PFA (10× magnification). Own data.

The big problem in organoid maturation is the formation of necrotic zones in the central part of organoids. In mature organoids, neurons are found only on the periphery (Figure 3). To overcome neuronal death, hyperoxygenation can be used, as in the protocol of Kadoshima et al. (2017). Additionally, neurotrophic factors such as BDNF are used for long-term organoid cultivation [41]. In some works, to prevent neuronal death in the central part, the authors focused their efforts on the cultivation technique. Some used spinner bioreactors [84] or multifluid bioreactors [50] to increase the diffusion rate of oxygen and critical metabolites in the organoid central part and prevent cell death. Most of these protocols allow the creation of electrophysiologically active organoids up to 5–6 mm in diameter with virtually no necrotic zones.

Some authors, on the contrary, show that stationary cultivation systems in suspension are preferable to dynamic ones [65,85]. They use simple bacterial culture dishes or plates and argue that this prevents mechanical damage to the cultured organoids from the bioreactor blades.

However, under both dynamic and stationary cultivation conditions, further organoid growth is inevitably limited by such physical factors as the diffusion rate of nutrients and oxygen into the spheroid and the removal of metabolic products. This problem has become even more critical due to the high standards of cell/tissue transplant material quality required for regenerative medicine.

### 3.3. Cultivation Time

The cultivation time is also an important parameter. Matsui et al. (2018) showed that organoids created using Lancaster protocol need long-term cultivation under dynamic conditions to reach maturity [42]. The authors found mature oligodendrocytes expressing myelin basic protein only after 6 months of cultivation.

Trujillo et al. (2019) demonstrated that long cultivation time is essential to make organoids possessing electrophysiological activity [86]. Using MEA, the authors show that neurons’ collective activity in cortical organoids becomes detectable only after 6 months of cultivation. They identified nonlinear “inverted-U” oscillatory subpeaks, indicating complex neuronal connection formation. The organoid neuronal network activity was remarkably similar to that of newborns detected by electroencephalography (EEG). Two- and four-month-old organoids did not have such activity, as shown by the absence of the subpeaks on MEA. Only single electrophysiological activity peaks were detected. Using immunocytochemical staining and functional analysis, the authors showed that in 6–10-month-old organoids, the content of GABAergic neurons increases, reaching 10–15% of the entire neuronal population, which is consistent with the situation in vivo.

In our opinion, to further improve the methods for creating functionally active organoids, the protocols for obtaining different types of neuronal cells in 2D cultures should be revisited. Today, these protocols are much more standardized and less time-consuming. However, it seems doubtful that it will be possible to shorten the differentiation time significantly.

### 3.4. Organoid Standardization

Despite significant progress in protocol development, the resulting organoids differ batch-to-batch [87]. There is significant variability in organoids created in different laboratories and/or cell lines. Unexpectedly, organoids made in the same laboratory and even of the identical iPSCs may vary dramatically. This variability limits organoid use in screening new therapeutic drugs or in preclinical studies, requiring high standardization. Several research groups published protocols for creating large quantities of standardized organoids for use in drug screening and electrophysiological studies [49,88,89].

Nevertheless, the standardization issue remains troublesome due to many steps in protocols, many influencing factors, and prolonged cultivation duration. For example, stem cells are susceptible to such physical conditions as temperature, the partial pressure of gases, osmotic pressure, etc. [90,91]. Therefore, it is necessary to control and standardize these parameters.

The heterogeneity of organoids also occurs due to non-standard cultivation components. This problem is most pronounced for Matrigel, serving as an extracellular matrix. Nasu et al. showed that Matrigel is essential for the correct organization of the neuroepithelium in vitro [92]. The matrix components stimulate migration, polarization, and the formation of neuroepithelial structures, “rosettes,” which are analogs of the neural tube. However, Matrigel is produced from the culture of tumor cells, and the final properties of Matrigel depend on cultivation and purification [93]. Synthetic hydrogels can be a solution to this problem [94]. For example, Qin et al. (2017) describe a protocol for creating brain organoids using microfibrils from sodium alginate and polyvinyl alcohol, serving as an extracellular matrix [95].

Another problem of standardization arises at the stage of saturation of spheroids with Matrigel or other matrices. The thickness of the matrix absorbed on the spheroid varies greatly. This variation can lead to significant differences in the gradient of oxygen, nutrients, growth factors, morphogens, or neurotrophic factors during subsequent cultivation. Thus, controlled methods for matrix saturation of spheroids [96] can help standardize brain organoids [97].

The issue of organoid standardization is also closely related to the problems of iPSC variability and standardization. The similarity of iPSCs to ESCs and cells of the inner cell mass and comparison of iPSCs derived from different somatic cell types were widely discussed in the literature for the past decade [98,99,100]. A detailed analysis of this topic is beyond the scope of this literature review. However, the individuality of iPSC clones and even variability depending on the number of cell culture passages should be considered.

The use of patient-specific iPSCs for organoid derivation does not allow controlling the influence of genetic variability. Therefore, there was a need to establish isogenic cell lines with the same genetic background, which differ only by the presence or absence of a specific mutation that causes pathology. These isogenic cells provide an “ideal” platform to study the molecular mechanisms of disease development, as well as for high throughput drug screening [101]. Isogenic brain organoids were used to model several diseases such as Alzheimer’s disease [57], Parkinson’s disease [54], and fragile X syndrome [102]. In our opinion, there is no need in isogenic systems to model diseases caused by mutations with complete penetrances, such as Huntington’s disease or early infantile epileptic encephalopathy 17. In such cases, the comparative study of iPSC-derived organoids from several patients and healthy controls will reveal the features of the disease that are inherent in all “diseased” organoids, regardless of their genetic background.

### 3.5. Organoid Quality Indicators

The researchers usually assess the quality of organoids, comparing their phenotype with brain structures. For this, histological and immunocytochemical methods are used. The omics methods are also used to assess gene expression and identify the cell composition of organoids. Besides, investigators pay more and more attention to physiological activities, particularly the electrophysiological activity that can be analyzed in living organoids.

The patch–clamp method can detect the membrane potential and obtain information about ion channel activity in an individual cell [103,104]. Even though the patch–clamp method is used to check ion channels in individual cells, the technique allows studying the emerging active cellular networks in organoids [105]. Using this technique, one can study molecular mechanisms of drug action, including therapeutic substances [106].

Optogenetic technologies based on light activation or suppression of genetically engineered ion pumps expressed in cells [107] also allow studying cells in organoids [108]. For example, Quadrato et al. showed an activity of photoreceptor cells and their ability to excite and inhibit neuron activity [41].

Additionally, scientists can assess neuron functionality via visualization of calcium waves. The calcium waves represent changes in fluorescence which derives from the binding of Ca^2+^ ions to genetically encoded reporters or small molecules [38]. Using specific fluorescent calcium indicators and confocal or multiphoton microscopy, Tukker et al. visualized calcium signals in neurons in response to various stimuli [48]. The calcium waves are visualized in small neuronal aggregates. However, it is impossible to analyze the entire neural network using visualization of calcium currents in organoids.

To analyze the entire neural network, researchers began to use MEA, recording electrophysiological signals from several sites [109,110]. This method shows the presence or absence of a formed working neural network in 3D organoids [39,111]. Several groups are working on 3D MEA methods [112] or a combination of MEA with calcium imaging methods [49], which will allow monitoring spontaneous or induced neural signals from several layers. Compared to other methods for detecting electrophysiological activity, there are fewer publications on the study of brain organoids using MEA. Nevertheless, MEA may become a critical test for organoid maturity.

An outstanding example of an integrated approach to the study of electrophysiological processes in brain organoids is the work of Zafeiriou et al. (2020) [113]. The authors worked on iPSC-derived cerebral organoids containing glutamatergic, GABAergic, catecholaminergic, and serotoninergic neurons, as well as myelinating (oligodendrocytes), non-myelinating (astrocytes) glial cells. Using the Fluo-8-AM calcium indicator, the authors demonstrated calcium waves and depolarization events by MEA. Adding GABA or glutamic acid caused depolarization events in young organoids. In mature organoids, glutamate but not GABA caused the depolarization. Thus, the authors observed the switch of excitatory to inhibitory GABAergic neurotransmission. This switch is a hallmark of neuronal network maturation in the developing brain.

The above approaches allow assessing the functional state of cerebral organoids and their maturity. Determining electrophysiological parameters of neurons in an organoid is necessary for quality control. Such tests may become mandatory in cell therapy to check the graft quality and transplantation readiness. In many neurodevelopmental disorders, synapses are not formed, or their functionality is impaired. Therefore, a functional test for comparing “disease” and “health” is needed. Additionally, neuron electrophysiological responses can become an excellent way for assessing putative drug effectiveness.

## 4. Approaches to Recreating Brain Complexity In Vitro

The embryonic brain is an extraordinarily complex structure with significant cellular and architectural diversity, increasing rapidly from the third week of pregnancy. Brain development requires the vascularization for oxygen and nutrient supply. In addition to various neurons of ectodermal origin, the embryonic brain contains cells of non-ectodermal origin—microglia, endothelial cells, and smooth muscle cells. Thus, researchers are working on different complex approaches to recreate the brain complexity in vitro.

### 4.1. Complex Organoids (Assembloids)

Several attempts were made to fuse spheroids or organoids of different cellular compositions to model more complex 3D brain structures (Birey et al. 2017; Bagley et al., 2017) [51,114]. For example, Birey et al. (2017) fused oligodendrocytes with an organoid consisting of GABAergic neurons and an organoid consisting of glutamatergic neurons and astrocytes [51]. Using these conglomerate structures, the authors demonstrated the migration of GABA neurons from the ventral to the dorsal forebrain, their integration, and the formation of cortical nerve chains. This process strikingly resembles events in embryogenesis.

Similarly, the complex organoids helped Xiang et al. (2018) simulate in vitro the development of the embryonic brain [115]. Xiang et al. (2018) merged an 18-day old cortical organoid with an organoid of the ganglionic eminence, a temporary structure present in the brain at the embryonic and fetal development stages. The ganglionic eminence is located in the ventral part of the telencephalon, extending into the ventricle cavity. The basal ganglia rudiment contributes to the development of a population of GABAergic interneurons of the cerebral cortex. Xiang et al. (2018) demonstrated the migration of neurons from the ganglionic tubercle to the cortical part of the conglomerate 2–3 weeks after organoid fusion.

Thus, complex organoids help model developmental processes and neurodevelopmental diseases associated with disruption of cell migration and cell connection. For example, complex organoids were used for modeling Timothy syndrome, caused by mutations in the calcium channel gene CaV1.2 [51].

### 4.2. Organoid Vascularization

Scientists try to develop protocols to produce vascularized organoids in vitro. In [116], using a combination of small molecules CHIR99021 and SB431542, as well as growth factors FGF2, VEGF, and BMP4, Wimmer et al. (2019) created vascular organoids that can form arterioles, capillaries, and venules in vivo. In another study, Pham et al. (2018) used a combination of cerebral organoids with endothelial cells previously obtained by separate protocols from the same iPSC line [117]. The authors placed non-vascularized 34-day-old cerebral organoids into Matrigel drops, previously inoculated with endothelial cells, incubated them for up to 54 days in a mixture of media for maturation cerebral organoids and for maintaining the growth of endothelial cells. They observed microvessels formation and growth deep into the organoid. Subsequent implantation of created organoids into brains of immunodeficient NGS (NOD/SCID) mice enhanced vascularization, as detected by immunocytochemistry with antibodies to human CD31. Shi et al. (2020) created vascularized cortical brain organoids by combining HUVEC endothelial cells with 35-day old non-vascularized organoids derived from iPSCs [118]. The authors had cultivated such organoids for more than 200 days without affecting the entire cell structure viability. They also showed that in vitro vascularization improves organoid cell maturation. They confirmed the electrophysiological activity of organoids using patch–clamp methods and calcium detection.

Cakir et al. (2019) [119] made vascularized cerebral organoids by combining the original and lentiviral transduced hESCs cells. Transduced cells expressed the ETV2 gene inducing differentiation into the endothelium under the control of a doxycycline-inducible promoter. The authors showed the high viability of the central zone cells in vascularized organoids by the patch–clamp method. By measuring the transendothelial resistance, they found a striking similarity to the blood–brain barrier physiological properties. Thus, by combining the protocols of neural and vascular differentiation, it is possible to obtain vascularized brain organoids (see also the recent review on brain organoid vascularization [120]).

### 4.3. Inclusion of Other Types of Brain Cells

Many researchers believe that fully functional neurons need to be adjacent to other types of cells in the correct spatial orientation [41,121,122]. Experiments on the co-cultivation of cortical neurons and astrocytes obtained from dissociated 45-day-old organoids have shown that the survival and growth of neurons require the presence of a microenvironment, at least glial cells [123].

Microglia cells, resident immune cells of the central nervous system, are used as the neuron microenvironment. These cells express various immunoactive molecules—IL1A, C1Q, TNF, etc., participating in controlling the neuronal viability and inflammatory reactions in vivo [124,125]. Several authors showed that the iPSCs differentiated into microglial cells capable of macrophage activity [126,127,128,129] supported the viability of the 2D neuronal culture. In vivo, microglia are involved in maintaining homeostasis and the formation of synaptic connections between neurons, utilizing “formative pruning” of synapses, and providing trophic support of neurons [130,131]). However, microglial cells’ characteristics are volatile in vitro, and they lose their morphology very quickly. One way to improve organoid quality is to assembly 3D cultures from cells of isogenic lines—neurons, glia, microglia, and astrocytes. For example, transcriptome analysis showed that, upon co-cultivation with neurons derived from iPSCs, microglia are closer in characteristics to those in vivo [132].

Despite all the efforts, the brain organoids remain a kind of Frankenstein. Although the organoids can be viable for a long time and be electrophysiologically active, their structure, activity, and cell composition are still far from the high complexity of the adult brain. To study the gene function and the molecular mechanisms of diseases, it is still good to combine organoid technology with mouse models and other methods, especially for late-onset and multifactorial diseases.

## 5. Conclusions

**Figure 4:**
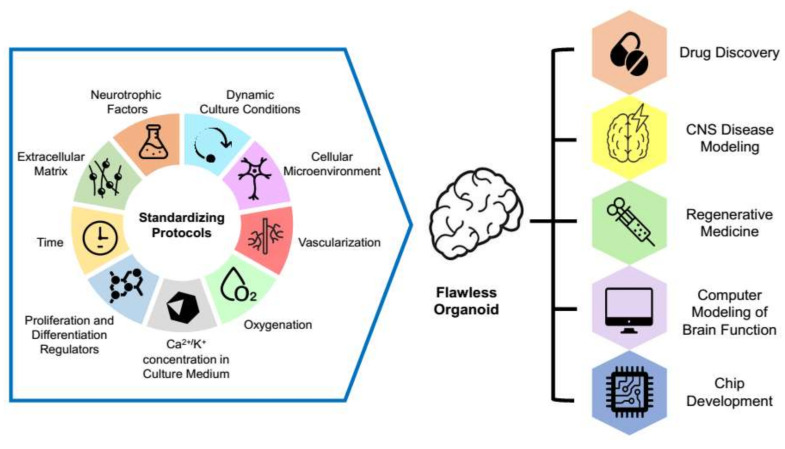
Current trends in development of cerebral organoid technology.

Overall, to obtain standard functionally active brain organoids, an integrated approach is required, which would be suitable for research purposes and the needs of regenerative medicine (Figure 4).

The standard protocol should include control over the physicochemical conditions of cultivation, e.g., a saturation of the media with oxygen, nutrients; a suitable instrument base to create dynamic conditions for cultivation; factors in the culture media: morphogens, growth factors, neurotrophic regulators, components of the extracellular matrix, and cellular microenvironment. Special attention must be paid to the timing and duration of factor application.

Currently, organoid technology is more reminiscent of the state of the art. Due to variability in organoid preparations, the same experiment must be conducted repeatedly to get reliable results. The poor organoid reproducibility limits their wide use for mass drug screening or biomedicine. However, combining further improvement of organoids creation and advances in research on natural and artificial biocompatible materials will allow modeling more complex systems and answering more interesting biological questions such as studying the mechanisms of interaction regulation between the nervous and other body systems: neuro–muscular, neuro–vascular, neuro–endocrine, etc.

## Data Availability

Not applicable.
